# 

**Published:** 1917-05

**Authors:** 


### OLD JOKES THAT WE HAVE MET.

---

Physician (looking into his anteroom where a number of patients were waiting): "Who has been waiting the longest?"

Tailor (who has called to present a bill): "I have, doctor; I delivered the clothes to you three years ago."—*London Evening Standard.*

---

A tramp knocked at a kitchen door and said: "Please, kind lady, I'm a sick man. The doctor gimme this medicine, but I need something to take it with."

The lady was ready to help.

"Poor fellow," she said, "do you want a spoon and a glass of water?"

The tramp answered: "No, mum, I wouldn't trouble you. But this medicine haster be took before meals. Have you got a meal handy?"—*Christian Herald.*

---

Girl: "I'm going to marry a doctor, so that I can be well for nothing."

Boy: "Why not marry a minister, so then you can be good for nothing."—*Ex.*

---

An old lady who had been introduced to a doctor who was also a professor in a university, felt somewhat puzzled as to how she would address the great man.

"Shall I call you 'doctor' or 'professor'?" she asked.

"Oh! just as you wish," was the reply; "as a matter of fact, some people call me an old idiot."

"Indeed," she said sweetly, "but then they are people who know you."—*Tit-Bits.*

---

A surgeon in a Western town, engaged to perform an operation of minor character upon a somewhat unsophisticated patient, asked him if he were willing to have only a local anesthetic.

"Sure," replied the other, "I believe in patronizing home industry whenever you can."

Is there any better or more logical standard by which to measure the progress of civilization than the number of things used to kill human beings?

Two or three thousand years ago, when everything was more or less crude, these were about all they had:

Bows, arrows, spears and catapults. Firebrands. Bludgeons. Stakes.

Now we have:

Machine guns. Bombs. Gas. Autos. Trolleys. Railroads. Electric wires. Grade crossings. Trusts.

Then, as now, however, they had medicine men.—*Life.*

---

A little boy was given too much underdone pie for his supper and he was soon roaring lustily.

His mother's visitor was visibly disturbed.

"If he was my child," she said, "he'd get a good, sound spanking."

"He deserves it," the mother admitted, "but I don't believe in spanking him on a full stomach."

"Neither do I," said the visitor, "but I'd turn him over."—*Success Magazine.*

---

Dr. A.: "Why do you always make such particular inquiries as to what your patients eat? Does that assist you in your diagnosis?"

Dr. B.: "Not that, but it enables you to ascertain their social position and arrange my fees accordingly."—*Topeka Journal.*

---

Little Tooty was up before the medical board for examination.

"Any special identification marks on the body?" asked the doctor.

"No, sir!"

"Here, wait a minute! What are all these bruises on the back of the neck?"

"Them? Oh, they're nothing! I've been subject to them for years! You see, I'm a clarinet player in an orchestra."

"I don't see how that can produce bruises on the back of your neck."

"It doesn't produce them exactly, but it places me in a position where I am very liable to get them."

"How is that?"

"I sit directly in front of the man who plays the slide trombone."

